# Diverse RNAs in human umbilical cord-derived exosomes and their therapeutic potential

**DOI:** 10.1080/15476286.2025.2589583

**Published:** 2025-11-12

**Authors:** Ali Khezrian, Zahra Sobhi Amjad, Armin Khaghani Boroujeni, Ali Shojaeian

**Affiliations:** a Research Center for Molecular Medicine, Institute of Cancer, Hamadan University of Medical Sciences, Hamadan, Iran; b Department of Microbiology, School of Medicine, Kermanshah University of Medical Sciences, Kermanshah, Iran; c Skin Disease and Leishmaniasis Research Center, Isfahan University of Medical Sciences, Isfahan, Iran

**Keywords:** HucMSC, exosome, miRNA, lncRNA, circRNA, therapeutic potential

## Abstract

Exosomes, nanosized extracellular vesicles (30–150 nm) secreted by various cell types, have emerged as crucial mediators of intercellular communication and promising therapeutic agents. This review highlights the diverse RNA cargo of exosomes derived from human umbilical cord mesenchymal stem cells (HucMSC-Exos), including mRNAs, miRNAs, long non-coding RNAs (lncRNAs), and circular RNAs (circRNAs), which regulate gene expression and cellular functions in target cells. The mechanisms of exosome biogenesis, release, and uptake are discussed, with emphasis on their ability to cross biological barriers such as the blood – brain barrier. HucMSC-derived exosomes exhibit therapeutic potential in wound healing, angiogenesis, neuroprotection, immunomodulation, and treatment of diseases like Parkinson’s, preeclampsia, and renal or hepatic injury. Specific exosomal miRNAs, such as miR-136, miR-335-5p, and miR-1246, demonstrate targeted effects. Additionally, exosomal RNAs show promise as disease biomarkers. Future directions involve standardization, targeted engineering, RNA profiling, clinical trials, and integration into personalized medicine strategies for regenerative therapy.

## 1. Introduction to exosomes

### 1.1. Definition and characteristics of exosomes

The origin story of EV research arguably goes back to Chargaff and West in their studies of blood coagulation in the 1940s. They discovered a ‘particulate fraction’ that sedimented at 31,000 g and had a high coagulation potential [1]. Then, 17 years later, Wolf described ‘a fine particulate matter, sedimentable by high-speed centrifugation, derived from platelets’ as ‘platelet round’, and a variety of terms were then used to describe the structures observed, including extracellular microvesicles, microparticles, small particles, and virus-like particles [2]. Trams and colleagues first coined the term ‘exosome’ in the context of EVs to describe vesicles that are produced directly by budding outward from the plasma membrane [3]. Later, Johnston used the term ‘exosomes’ to describe the vesicles released after fusion of MVBs with the plasma membrane [4]. Experiments in which EVs were specifically identified as biological entities with enzymatic and functional potential then began in the 1980s and 1990s [5]. Exosomes are spherical, nanoscale vesicles (30–150 nm) [6] with two lipid layers composed of sphingomyelin, ceramide, cholesterol, phosphatidylcholine, and phosphatidylethanolamine [7–9]. Almost all cell types, including normal and sick cells, secrete them, and they can be found in a wide range of biological fluids and tissues, such as blood, saliva, fat, and amniotic fluid [10]. They have been proven, for example, to be required for embryonic development and to have immunomodulatory properties. Furthermore, they promote wound healing and tissue regeneration. EVs have an important role in neurological illnesses, induce carcinogenesis and metastasis, and enhance viral particle transmission and spread in certain diseases [11–14]. The three main subtypes of vesicles are microvesicles (MVs), exosomes, and apoptotic bodies, which are distinguished based on their biogenesis, release pathways, size, content, and function [15]. In reality, the density of the vesicle varies from one to the next and is regulated by the protein-to-lipid ratio [16]. When combined, these factors show that exosome density, size, and shape are not characteristic properties of exosomes, but rather highly variable consequences that are mostly governed by the unique protein, lipid, enzymatic, and mineral content of each individual exosome [17,18]. A wide range of surface proteins, including integrins, major histocompatibility complex (MHC) I and II, flotillin-1, tetraspanins (CD9, CD63, and CD81), Hsp70, TSG101, Alix, and others, have been implicated as exosome markers and have been implicated in exosomal adhesion. Furthermore, exosomes include other proteins, numerous RNA types, and DNA [19]. An important point about exosomes is that they retain their metabolic activity even after months of storage at −70°C, so they can be well stored for future use [20]. For this reason and because of their extensive content, researchers are constantly researching the idea of employing EVs as carriers to precisely deliver drugs and biologics to sick tissue cells [11,21]. The aim of our study is to provide a brief overview of the current knowledge on exosome synthesis, molecular characterization, and functional activities of exosomal RNAs in cell contacts. Finally, we studied HucMSC-Exos and their RNA content, and investigated the therapeutic potential of modified exosomes as targeted drug delivery systems, as well as their viability as clinical biomarkers.

### 1.2. Biogenesis

MSCs produce exosomes in response to paracrine stimulation [22]. Their biogenesis begins with inward budding of the plasma membrane, which results in the creation of intermediate endosome-vesicles, the multivesicular bodies (MVBs) [23]. The biogenesis process distinguishes between exosomes, microvesicles (MVs) and apoptotic bodies [24]. Cells undergoing programmed cell death and signal cell engulfment release apoptotic bodies; MVs are generated by budding and blebbing from the plasma membrane; and exosomes are of an endocytic origin [14]. Exosomes are synthesized and secreted in four phases. Phase I is when the membrane of the multivesicular body (MVB) buds inward to form the exosome. Phase II is the initial stage of endosome formation within the cell. Late endosome development take place during Phase III. And phase IV is associated with exosome secretion [25]. MVBs can have one of two final fates: the exosome can undergo fusion with lysosomes or be released as the extracellular discharge of internal cargos into the extracellular area after fusion with the plasma membrane (PM) [25,26]. The endosomal sorting complexes required for transport (ESCRT) regulates the synthesis of multivesicular endosomes, but other pathways may possibly be involved. By detecting ubiquitinated membrane proteins, the ESCRT complex enhances their import into the multivesicular endosome [27]. Before exosomes are released, a number of biological events must occur, including the creation of intraluminal vesicles (ILVs) in MVBs, MVB transit to the plasma membrane, and MVB fusion with the membrane [28]. The word exosomes refers to ILVs that are discharged into the extracellular space after fusing with the plasma membrane ‎ [14]. Exosomes are commonly identified based on their size and expression of exosome flag proteins such as CD63, CD81, and CD9 [19].

### 1.3. Exosome delivery to cells: therapeutic applications and biological impacts

Exosomes are capable of a number of functions. Because of their natural origin, capacity to mediate intercellular communication, and ability to encapsulate a variety of biological components, including proteins and nucleic acids, within the lumen or lipid bilayer membrane, exosomes are being utilized more and more as therapeutic drug delivery vehicles [29]. These functional extracellular vesicles transport a complex cargo of proteins, lipids, cholesterol, and various cell lines containing RNA molecules, particularly Messenger RNA (mRNA) and microRNA (miRNAs), as well as other non-coding RNAs (ncRNAs) that can either translate into proteins or control protein expression in the recipient cells [30–32]. Exosomes are thus a novel kind of intercellular communication. Exosomes may potentially have a function in signal transduction, immune response, and antigen presentation [32]. There have been numerous studies on exosome-mediated cell-to-cell communication. The following methods enable target cells to ingest the contents of exosomes: (i) interaction between target cell receptors and exosome membrane proteins; (ii) interaction between soluble fragments generated by exosome membrane proteins and cell surface receptors; and (iii) internalization of exosome contents [33].

Exosomes have substantial benefits over alternative delivery techniques, such as liposomes and polymeric nanoparticles [34]. Exosomes interact with cells, participate in the antigen presentation cascade, and are linked to a range of important immunologic processes, including immune surveillance [35]. Recent studies have drawn attention to the critical usage of exosomes as personalized, targeted medicine delivery systems [23,36,37].

Today, the parameters that must be met before any type of vesicle can be employed for targeted medicine administration are well established. These requirements include: (I) the vesicle to be biocompatible, meaning non-toxic and non-immunogenic; (II) the ability to evade macrophage uptake and circulate for extended periods of time in order to reach their cellular targets; (III) the ability to maintain stability (i.e. size, structure, and drug load) during blood stream circulation prior to reaching the therapeutic target in the body; and (IV) the ability to load the vesicle with enough drug to elucidate a therapeutic response [23,38]. These approaches are often used to deliver compounds from various medication classes, including anticancer, antifungal, and analgesics [39]. Since the majority of the cells’ exosomes are intrinsic carriers of endogenous protein molecules, the exosome carrier system may work well for delivering peptides or proteins [25]. Exosomes are outstanding and excellent drug delivery vehicles for use in cancer therapy and other disorders because of their biocompatibility, stability, preferential tumour homing, and varied targeting efficacy [40]. Numerous studies have revealed that exosomes can pass the blood-brain barrier (BBB), allowing various environmental substances to reach the central nervous system (CNS) [41–43]. ‎ Therefore, exosomes and their physiologically active cargos may offer potential therapeutic roles and predictive information for a range of ailments, including cancers [44], lipid metabolic disorders [45], neurological disorders [46], cardiovascular and renal diseases [47,48], and chronic inflammation [31].

## 2. Exosomes derived from human umbilical cord (HucMSC-Exos)

Human umbilical cord mesenchymal stem cells (HucMSCs) can be obtained using a non-invasive process and are similar to MSCs from bone marrow in that they exhibit high self-renewal, proliferative and migratory ability of skin cells, angiogenesis ability, and minimal immunogenicity [22,49,50]. These properties may make HucMSCs more beneficial than MSCs derived from other sources for cell transplantation therapy, as they are easier to collect and more abundant than other MSC sources, reducing ethical controversies [51]. HucMSC-Exos, have been proposed as a potential therapeutic strategy for tissue regeneration and repair [52,53].

**Figure 1.**
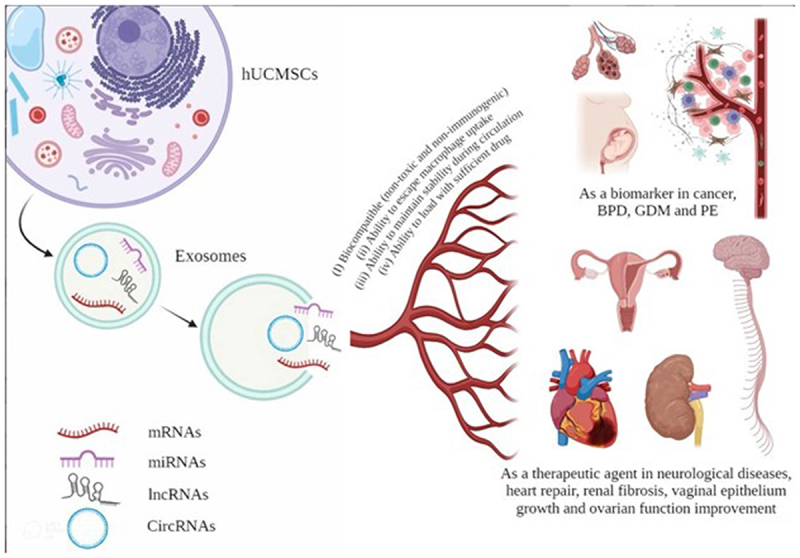
HucMSC-Exos containing diverse rna cargoes and their effects. Exosomes contain a rich collection of RNAs, including mRNAs, miRNAs, long non-coding RNAs (lncRNAs) and circular RNAs (circRNAs), which play pivotal roles in the regulation of gene expression and cellular function. These vesicles have a natural ability to cross biological barriers (I) biocompatibility of the vesicle, meaning non-toxic and non-immunogenic. (ii) the ability to evade capture by macrophages and circulate them for long periods of time in order to reach cellular targets. (iii) the ability to maintain stability (eg, size, structure, and drug load) during circulation prior to reaching the therapeutic target in the body; and (iv) the ability to load the vesicle with sufficient drug to elicit a therapeutic response. Their effects have been shown in wound healing, angiogenesis, neuroprotection, immune modulation. They can act as a biomarker in cancer, bronchopulmonary dysplasia (BPD), gestational diabetes (GDM) and preeclampsia (PE) and as a therapeutic agent in neurological diseases, heart repair, renal fibrosis, vaginal epithelium growth and ovarian function improvement.

HucMSC-Exos have been shown to protect against acute tubular injury and reduce cardiac ischemia/reperfusion damage, highlighting their potential as a novel therapy strategy for a variety of illnesses [54–56]. According to Tingfen Li et al., human bone marrow MSCs (BMSCs) and HucMSCs may play a role in the healing of injured livers and kidneys [51]. HucMSC-Exos have shown anti-inflammatory effects in the treatment of a variety of diseases. It is still unclear if they can protect neurons affected by Parkinson’s disease by suppressing inflammatory processes mediated by microglia [57]. Compared to normal MSCs, HucMSCs release more wound healing factors. HucMSCs promote fibroblast migration, proliferation, and collagen synthesis [33]. Surprisingly, a number of studies show that MSCs-Exos have been linked to an acceleration of wound healing [58–60]. For example, Linlin Liang and colleagues found that injured endometrial epithelial cells (EEC) after oxygen and glucose deprivation/reoxygenation (OGD/R) treatment with exosomes produced by HucMSCs showed improved viability and reduced cell death, and anti-inflammatory and repair effects [61]. Jinwen Liu et al. used a second-degree deep burn injury mouse model to investigate the role of HucMSC-Exos in angiogenesis and skin wound healing in vivo. The results revealed that HucMSC-Exos enhance the rate of wound closure, increases CD31 expression in the body, and accelerate wound healing and skin angiogenesis by delivering angiopoietin-2 [62]. Studies demonstrate that exosomes released by MSCs activate a number of signalling pathways that enhance cell proliferation and wound healing [63,64]. Yoon-Jin Kim’s study’s findings indicated that HucMSC-Exos are absorbed by human skin, increases the skin’s creation of collagen I and elastin-two proteins required for skin rejuvenation – and shows the possibility of combining HucMSC-Exos with cosmetic or therapeutic materials [33]. Furthermore, Mei-ting Chen’s findings indicated that HucMSC-Exos enhance insulin sensitivity in human adipocytes that are insulin resistant [65]. According to Li Sun et al., HucMSC-Exos protected rats from weight loss without causing any adverse effects on liver or renal function. Additional methods of detection, including pyrogen, haematology indices, haemolysis, vascular and muscle stimulation, and systemic anaphylaxis, showed that HucMSC-Exos were well tolerated in animal models [20]. Kun Xie et al. found that via modulating the glycogen synthase kinase-3β (GSK3β) driven Wnt/β-catenin signalling pathway, HucMSC released exosomes carrying miR-1246 to hepatocytes, providing a protective effect against hepatic ischemia/reperfusion damage (IRI) [66]. Another study investigated the effect of HucMSC-Exos in improving the ability of aged bone marrow MSCs (OMSCs) to repair the heart by transfecting exosomal miR-136 and reducing Apaf1 expression, and found that exosomes from young mesenchymal stem cells could enhance the function and activity of aged MSCs for heart repair [67] (Figure 1).

## 3. Diverse rna contents of HucMSC-Exos

The diverse RNA biotypes found in EVs reflect the donor cells’ kind and physiological/pathological state, as well as their RNA content. For example, one study found that the abundance of AGO2-related miRNAs in extracellular vesicles is enhanced in the cell culture supernatant of cancer cells overexpressing the KRAS oncogene [68].

Several techniques, such as size exclusion chromatography, asymmetric field-flow fractionation, high-resolution density gradient centrifugation, and immunoaffinity purification, were used to address the issue of how different separation techniques affect the RNA profiles of extracellular vesicles. EVs have been shown to be useful in transporting biological macromolecules, including nucleic acids, to recipient cells in a customized manner without alerting the immune system [69].

Their tiny size, abundance, ability to communicate with membranes, and cytoplasmic location (rather than the nucleus) all contribute to their correct affiliation with EVs. However, the packaging of RNA in these vesicles is highly complex and involves a variety of mechanisms. EVs, for example, use viral-like Gag proteins on the inner plasma membrane to bind to RNA and emerge as virus-like particles from the cell membrane [69,70].

In contrast, the majority of RNAs are transported in association with RNA-binding proteins (RBPs). A significant subset of these proteins, known as heterogeneous nuclear ribonucleoproteins (hnRNPs), appear to be in the centre of vesicular trafficking and RNA post-transcriptional regulation [70]. For example, hnRNPK is directly involved in LC3-dependent EVs loading and secretion [71].

Several more RBPs with affinity for binding sites for certain RNA sequence motifs have been identified. Additional processes that effect RNA binding, stability, miRNA translation, and biogenesis include specific RNA sequence motifs and/or secondary configuration, as well as RNA and/or RBP modifications such as ubiquitylation, sumoylation, phosphorylation, and uridylation [72]. As a result of its ability to influence the functional features of recipient cells, RNA distribution in extracellular vesicles has the potential to serve as a new biomarker and therapeutic technique for a wide range of disorders. These RNAs include mRNA, miRNA, long noncoding RNA (lncRNA), and circular RNA [69,73]. lncRNAs and circRNAs are two important categories of big ncRNAs; they are transcribed from the genome but lack protein-coding capability; yet, they play many functions in various cell processes [74].

DNA transcription produces mRNA, which is responsible for protein synthesis. In eukaryotes, pre-mRNA requires processing and maturation to remove introns and link exons together [75]. A 7-methylguanosine cap is then added to the 5’ end, and a poly (A) tail, a sequence of adenine nucleotides, is added to the 3’ end of the RNA transcript, therefore improving its stability and transport. MiRNAs, which originate primarily in the intron region, also play an important role in gene regulation. Their binding to the 3’UTR regions of mRNA is likely to repress translation, whereas their binding to promoter regions increases transcription, but this is not always the case [76].

NcRNAs are involved in DNA synthesis and genome rearrangement, and they influence gene expression during transcription, RNA processing, and translation [77]. Long non-coding RNAs (lncRNAs) are a diverse group of regulatory transcripts. CircRNAs have tissue and cell-specific expression patterns, and their synthesis is carried out via backsplicing. Both are normally modest, but strongly expressed in certain cell types, tissues, developmental stages, and disease states [78]. Initially, it was found that mRNAs and miRNAs exist in exosomes and can be transferred to other cells and have functional roles [79].

Various RNAs are found in EVs and act as genetic cargo in a number of physiological and pathological processes, according to research [80].

For this reason, various studies [81–83] investigated the changes of these RNAs in HucMSC-Exos so that they can be used as molecular markers for clinical diagnosis and treatment of various pathologies [84].

Wang et al. found 317 circRNAs, 104 lncRNAs, and 135 mRNAs changed in the cord blood of infants with bronchopulmonary dysplasia (BPD). The pathways for transport and catabolism, signal transduction, translation, immune system, and carbohydrate metabolism showed the most substantial expression alterations. GO and KEGG analysis to further investigate the role of these RNAs in different studies showed that they are involved in the growth of endothelial or epithelial cells and with various processes including angiogenesis, mammalian target of rapamycin (mTOR) signalling pathway, Wnt signalling pathway, resistance to epidermal growth factor receptor tyrosine kinase inhibitor, and altering growth factor beta receptor signalling [83].

Meanwhile, the mTOR signalling pathway was shown to have two protein complexes with distinct functions, namely complex 1 (mTORC1) and complex 2 (mTORC2). mTORC1 consists of mTOR, mTOR-related protein, LST8 homolog (mLST8), raptor, and two repressors (PRAS40 and DEPTOR). Inhibition of mTOR regulatory protein, a major subunit of mTORC1, increased autophagy and attenuated apoptotic death, suggesting that differentially expressed exosomal RNAs may play an important role in BPD through the mTOR signalling pathway. Molecular functions revealed that the possible role of exosomal hsa_circ_0086913 is important in BPD and potentially interacts with hsa-miR-330-5p, hsa-miR-4656, hsa-miR-6829-3p, hsa-miR-103a-3p, hsa-miR-107, hsa-miR-4688, hsa-miR-7161-3p, hsa-miR-3192-5p, hsa-miR-3620-5p, hsa-miR-4656, hsa-miR-1182, hsa-miR-4656, hsa-miR-4688 and hsa-miR-6783-3p. Also, the expression level of hsa-miR-103a-3p related to the phosphatidylinositol 3-kinase/protein kinase B (PI3K/Akt) signalling pathway was decreased in cord blood-derived exosomes from the BPD group compared with the NBPD group, which is significantly involved in cell proliferation, cell migration, and tube formation. In addition, the expression level of TM4SF1, which promotes angiogenesis through the Akt signalling pathway, is a potential target gene of hsa-miR-103a-3p and was increased in cord blood-derived exosomes from infants with BPD. These results indicated that the hsa_circ_0086913/hsa-miR-103a-3p/TM4SF1 interaction network is likely to play a role in the development of BPD [83].

Another study looked into the involvement of various RNAs derived from HucMSC-Exos in the development of gestational diabetes (GDM) and foetal growth, and discovered that exosomes differed in form and composition between healthy and diseased groups. Microarray study, for example, revealed that exosomal TIMM8B, SIRT1, and MIEF2 expression was dramatically elevated, while exosomal ABCD1 expression was significantly reduced in GDM samples. This demonstrated that all exosomal mRNAs, lnCRNAs, and circRNAs of umbilical cord blood regulate the metabolic process, growth, and development [82,85].

Several pathways are significantly closely related to metabolic, developmental and growth processes that are important in the development of GDM and foetal growth, such as the pentose phosphate pathway, cholesterol metabolism, galactose metabolism, cellular processes, DNA replication, RNA transfer, etc. As we know, exosomal circRNAs regulate gene expression through the miRNA sponge mechanism and may reduce the inhibitory effects of miRNAs on target molecules, thereby regulating gene expression. For example, tumour-released exosomal circ-PDE8A promotes invasive growth through the miR-338/MACC1/MET pathway in pancreatic cancer. Also, gastric tumour-derived exosomal circRNA promotes browning of white fat by targeting the miR-133/PRDM16 pathway. In this study, miR-330, miR-23a, and miR-16-5p were found to be upregulated in plasma of GDM patients and corresponded to circ_0092108, whose downregulation was confirmed in cord blood exosomes of GDM patients. Therefore, we speculate that the role of circRNAs in GDM may be related to miRNA-mediated effects [82].

Microarray analysis revealed that the HucMSC-Exos of PE patients included 143 up-regulated circRNAs and 161 down-regulated circRNAs when compared to the control group. Cao et al. investigated the role of circRNAs from HucMSC-Exos in the development of preeclampsia (PE). According to the study’s findings, circRNAs with varied expression levels influence metabolic processes, trophoblast growth and invasion, and are important for the PI3K-Akt signalling pathway, all of which have a significant impact on the development of PE. The results showed that miR-17-3p and miR-424-5p corresponded to circ_0077260, whose increased expression was confirmed in cord blood exosomes of preeclamptic patients; miR-197-5p and miR-431-5p potentially bind to circ_0076206 with downregulation; while miR-424-5p and miR-483-3p potentially corresponded to circ_0090100 with upregulation. In particular, exosomal miR-486–1-5p and miR-486–2-5p have been reported to be upregulated in preeclamptic pregnancy compared with normal pregnancy. And miR-486-5p corresponded to downregulated circ_0076206. Therefore, it was found that the roles of altered exosomal circRNAs are related to metabolic processes, trophoblast development, and invasion-related signalling pathways, which may be related to miRNA-mediated effects [81].

Given the diverse roles of exosomes in local and distant cellular communication, cell-secreted and engineered exosomes have shown promising therapeutic potential in studies. Importantly, exosome-secreting cells can be modified to enhance the therapeutic properties of their exosomes, either by exposing the cells to cytokines or by gene transfer into exosome-producing cells. For example, mouse bone marrow-derived dendritic cells treated with recombinant murine IL-10 release exosomes that have enhanced immunosuppressive effects. The cargo of MSC-Exos contains over 850 proteins including growth factors, angiogenic factors, and factors that promote bone formation in situ. miRNAs also play important roles, including miRNA-126 and miRNA-224-3p, which have been associated with the angiogenic effects of MSC-Exos. miR-144, miR-21-5p, and miR-19a are involved in tissue regeneration by stimulating cell proliferation and reducing apoptosis, which is mediated through downregulation of PTEN, which increases the Akt signalling pathway for survival and leads to decreased levels of pro-apoptotic caspases-3, −8, and −9. Furthermore, MSCs exosome-delivered miR-100-5p targets mTOR mRNA, which enhances autophagy in osteoarthritis and leads to bone growth. miR-223 also has immunomodulatory properties and resulted in decreased expression of Sema3A and Stat 3, which inhibited the inflammatory response in macrophages and reduced cardiomyocyte death during sepsis [86].

EVs generated from ADSCs play a greater role in angiogenesis and adipogenesis than other exosomes, as evidenced by RNA sequencing and bioinformatic analyses (e.g. HES1, CEBPα, TCF4, KLF7, TGF-β). For example, two let-7 family miRNAs (let-7i-5p and let-7f-5p) generated by ADSCs stimulate angiogenesis. Furthermore, EV-derived lncRNAs, such as MALAT1 and H19, have been associated to apoptosis, adipogenesis, carcinogenesis, and cell proliferation. Exosomes released from adipocytes have also been found to trigger circ-deubiquitination (circ-DB) transfer, which then suppresses miR-34a and activates the USP7/cyclin A2 signalling pathway in the case of circRNAs [87].

Some research also looked into the therapeutic potential of HucMSCs-Exos and the RNAs involved. For example, a study looked into whether exosomes obtained from hUMSCs may boost the activity of BMSCs from elderly people (OMSCs) and improve their ability to heal the heart. OMSCs have considerably increased activity of senescence-related factors, including β-galactosidase, p53, p21, and p16, compared to HucMSCs. Treatment with HucMSC-Exos drastically reduced OMSC senescence characteristics while increasing their proliferation, migration, differentiation, and anti-apoptotic activity. These findings are related to the production of miR-136 in UMSCs, which inhibits the apoptosis peptidase activating factor (Apaf1), the miR’s downstream gene. Also, mouse studies demonstrated greater cardiac function, reduced fibrosis, and more angiogenesis after treating OMSCs with UCMSC-Exos in mice after heart attack [67]. This is consistent with previous evidence that maternal and umbilical cord exosomes contain a set of miRNAs that are strongly involved in the angiogenesis process [88].

The effect of exosomes formed from HucMSC-Exos on vaginal epithelium (VK2) was examined in order to test the idea of their participation in epithelial cell proliferation. The results indicated that HucMSC-Exos enhanced the cell cycle and inhibited apoptosis through miRNAs involved in the processes of cell cycle, development, and differentiation, such as miR-100, miR-146a, miR-21, miR-221, and miR-143, and significantly increased VK2 growth after treatment in a dose-dependent manner [89] (Table 1).

**Table 1.** Functions of HucMSC-Exos dependent on their rna contents in different studies.

Functions	Finding	Reference
Therapeutic	HucMSCs-Exos can enhance the activity of OMSCs due to the expression of miR-136, reducing senescence phenotypes and improving their capacity for cardiac repair.	Zhang et al., 2020 [67]
	HucMSCs-Exos significantly enhance HUVEC proliferation, migration, and tube formation due to the expression of a subset of migration-related microRNAs, including miR-210-3p, miR-376c-3p, miR-151a-5p, miR-296-5p, miR-122-5p, and miR-550a-5p.	Jia et al., 2018 [88]
	HucMSCs-Exos promoted cell cycle progression and inhibited apoptosis through miRNAs involved in cell cycle, development and differentiation processes including miR-100, miR-141a, miR-2. 221 and miR-143. VK2 growth was significantly increased after treatment in a dose-dependent manner.	Zhu et al., 2019 [89]
	HucMSCs-Exos ameliorate inflammation and EMT phenotype of HK-2 cells, reduce ADAM19 protein levels, and play an important role in reducing fibrosis and kidney injury.	Qiu et al., 2022 [90]
	HucMSCs-Exos improved follicle numbers and hormonal levels and improved ovarian function in mice with NOA due to inhibition of PTEN expression and suppression of apoptosis by miR-21-5p.	Li et al., 2023 [91]
	Reversal of glucocorticoid-induced osteoporosis (GIOP) using HucMSC-Exos through the PI3K/AKT signaling pathway	Zhao et al., 2025 [92]
	Inhibition of BMSCs apoptosis and prevention of disuse-induced osteoporosis (DOP) in rats by HucMSC-Exos via the miR-1263/Mob1/Hippo signaling pathway	Yang et al., 2020 [93]
	Effective treatment of skin wounds using HucMSCs-Exos by inhibiting the nuclear translocation of apoptosis-inducing factor (AIF)	Zhao et al., 2020 [95]

Qiu et al. found that HucMSC-Exos play an important role in reducing kidney injury and fibrosis. Exosomal miRNAs regulate TGF-β1-induced EMT in human renal tubular epithelial cells (RTECs). Transfecting miR-335-5p, which decreases ADAM19 protein expression, increased the EMT and inflammatory phenotype of TGF-β1-induced HK-2 cells. This presents a promising therapeutic method and targets for the clinical treatment of renal fibrosis [90].

To restore the function of ageing ovaries, the therapeutic efficacy of HucMSC-Exos was tested in a mouse model of normal ovarian ageing (NOA). Treatment with HucMSC-Exos in mice with NOA increased the number of follicles and hormone levels, as well as enhanced ovarian function. This activity was caused by the regulation of PTEN expression and the reduction of apoptosis by miR-21-5p, indicating that MSC-Exos transplantation is potential as a therapeutic against ovarian ageing [91].

A study showed that glucocorticoid-induced osteoporosis (GIOP) was reversed using HucMSC-Exos through the PI3K/AKT signalling pathway and inhibition of lipid peroxidation [92].

Disuse-induced osteoporosis (DOP) is a common complication caused by lack or no use of mechanical loading and has not been satisfactorily treated. Yang et al. studied the apoptosis of BMSCs in rat DOP by HucMSC-Exos. RNA-seq and q-PCR results showed that among the differentially expressed microRNAs, miR-1263 was the most abundant, which bound to Mob1 and could activate YAP expression by inhibiting it. Hippo inhibition reversed the effect of HLU-induced apoptosis on BMSCs in vitro. The results showed that HucMSC-Exos were effective in inhibiting BMSCs apoptosis and preventing rat DOP through the miR-1263/Mob1/Hippo signalling pathway [93].

Zhang et al. also evaluated the effect of HucMSC-Exos on microglial-induced neuroinflammation after ischaemic stroke. The results showed that injected HucMSC-Exos were able to reach the site of ischaemic injury and could be internalized by cells both in vivo and in vitro. In vitro, treatment with HucMSC-Exos reduced microglial-induced inflammation after oxygen-glucose deprivation (OGD). In vivo results showed that treatment with HucMSC-Exos significantly reduced infarct volume, attenuated behavioural deficits, and improved microglial activation, as measured three days after transient cerebral ischaemia. Furthermore, inhibition of miR-146a-5p (miR-146a-5p k/d Exos) partially reversed the neuroprotective effect of HucMSC-Exos. A mechanistic study revealed that miR-146a-5p in HucMSC-Exos attenuates the neuroinflammatory response mediated by microglia through the IRAK1/TRAF6 pathway. These results elucidate the potential therapeutic mechanism of MSC function and provide evidence that HucMSC-Exos is a potential cell-free therapeutic option for ischaemic stroke [94].

Zhao et al.‘s study on the use of mesenchymal stem cell-derived exosomes in skin wounds showed that treatment with HucMSC-Exos significantly increased the proliferation and migration of HaCaT cells in a time- and dose-dependent manner, suppressed H2O2-induced HaCaT apoptosis by inhibiting the nuclear translocation of apoptosis-inducing factor (AIF), and increased the expression of poly ADP ribose polymerase 1 (PARP-1) and poly (ADP-ribose) (PAR). Animal experiments showed that compared with HucMSCs, HucMSC-Exos reduced full-thickness skin wounds by increasing epidermal re-epithelialization and dermal angiogenesis. These findings indicated that direct administration of HucMSC-Exos may effectively treat skin wounds and could be of great value in clinical settings[95].

### 3.1. Clinical trials

Clinical trials in this field have mostly not reached a final conclusion, but we will mention a few of them. The clinical trial examined the administration of EVs derived from acellular cord blood MSCs (CF-CB-MSCs-EVs) in forty patients with chronic kidney disease (CKD) stage III and IV, in which the patients were divided into two groups; twenty patients as treatment group A and twenty patients as matched placebo group B. Two doses of MSCs-derived EVs were administered to patients in group A. Blood urea, serum creatinine, urine creatinine albumin ratio (UACR) and estimated glomerular filtration rate (eGFR) were used to assess kidney function and TNF-α, TGF-β1 and IL-10 were used to assess the improvement of inflammatory immune activity. The results showed that participants in group A showed significant improvements in eGFR, serum creatinine level, blood urea, and UACR, indicating that the administration of CF-CB-MSCs-EVs is safe and can reduce the inflammatory immune response and improve overall renal function in patients with CKD grade III-IV [96].

Clinical trials coded NCT05499156 investigated the immune-modulating effects of HucMSC-Exos on Crohn’s disease with refractory perianal fistulas. This is an ongoing, prospective, single-arm, phase 2 clinical trial. The study enrolled patients with Crohn’s disease with active perianal fistulas who had failed at least one course of anti-TNF therapy and had not received any therapy in the previous six months. Results showed that a total of 20 patients were eligible for this phase of the study, of whom 13 completed the 6-month follow-up period and were included in the final analysis. A total of 27 fistula tracts were observed in these patients. Eight patients (61.5%) were completely closed; one patient (7.6%) did not respond to treatment at all. Four patients (30.7%) who had multiple fistulas had some of their ducts resolved. Of the 27 fistula ducts, six patients (22.2%) remained active. All patients reported a decrease in discharge volume. No sequelae or adverse events were observed [97]. Table 2 lists some clinical trials that are currently underway.

**Table 2.** Clinical trials of treatment with HucMSCs-Exos.

No	Clinical trial ID	Study title	Disease	Phase	Country
1	NCT02138331	Effect of microvesicles and exosomes Therapy on β-cell Mass in Type I Diabetes Mellitus (T1D)	T1D	2/3	Egypt
2	NCT03437759	MSC-Exos promote healing of MHs	Macular degeneration	1	China
3	NCT04213248	Effect of UCMSC-Exos on dry eye in patients with cGVHD	Dry eye	1/2	China
4	NCT05871463	Effect of MSCs-Exos in decompensated liver Cirrhosis	liver cirrhosis	2	Iran
5	NCT05413148	The effect of MSCs and MSCs- Exos on visual functions in patients with retinitis pigmentosa	Retinitis pigmentosa	2/3	Turkey
6	NCT05669144	Co-transplantation of MSCs-Exos and autologous mitochondria for patients candidate for CABG surgery	Myocardial infarction	1/2	Iran
7	NCT04356300	HucMSCs-Exos for multiple organ dysfuntion syndrome after surgical repaire of acute type A aortic dissection	Aortic dissection	N/A	China

### 3.2. Challenges of using exosomes in research

The favourable properties of exosomes, as natural extracellular vesicles, in delivering bioactive compounds, penetrating biological barriers, and selectively targeting specific cells with low immunogenicity, have made them candidates for therapeutic and diagnostic applications and have been widely studied; however, challenges still remain [98].

However, their clinical application faces significant obstacles, including rapid clearance from the circulation, heterogeneity in cargo and biomarkers, and limitations in isolation, storage, and scalability. Although no technology can currently completely isolate and purify exosomes, there are still some practical isolation and purification techniques based on exosome properties. 1) Density gradient separation increases the purity of exosomes by further separating particles based on density and effectively removing non-exosomes. 2) Size exclusion chromatography separates exosomes through a stationary phase consisting of a porous polymer and is mainly used to isolate exosomes from plasma; 3) Immunological separation is based on the immunological affinity interactions between proteins in the membrane of exosomes and their antibodies. So far, the most common method for exosome isolation is ultracentrifugation, which is complex, tedious, and time-consuming. Recently, a novel alternating current electrokinetic microarray chip device was demonstrated that makes exosomes isolation convenient and rapid. It also makes exosome analysis suitable for point-of-care diagnostic applications. Advanced isolation processes and ideal growth conditions have increased yield and purity, while novel storage tactics such as cryoprotectant freezing and lyophilization offer viable options for long-term stability and delivery [99].

Exosomes derived from different cells may have different targets. Indeed, the mechanism of exosomes and their target cell surface receptors deserves in-depth study. In this context, surface modifications (e.g. pegylation and ligand engineering), encapsulation in hydrogels or nanoparticles, and genetic engineering of exosome-producing cells have all shown promise in improving exosomal retention, targeting specificity, and therapeutic efficacy [98].

Finally, the current understanding of the physiology, diversity, internalization, and molecular transport of exosomes is too limited to draw precise conclusions about the mechanisms by which exosomes interact with and modify recipient cells, and it is necessary to continue to address scalability issues and define standards for isolation, storage, and distribution to ensure reproducibility and consistency in clinical applications. Integrating AI and computational modelling into exosome design and prediction could revolutionize regenerative medicine, oncology, and beyond, bridging this gap between research and application.

## 4. Conclusion and future direction

Exosomes, particularly those generated from HucMSCs, have shown promise in regenerative medicine and targeted drug delivery. This study focuses on the heterogeneous RNA composition of these exosomes, which includes mRNAs, miRNAs, lncRNAs, and circRNAs, as well as their functions in many physiological and pathologic processes. The unique makeup of HucMSC-Exos, their capacity to pass biological barriers such as the blood-brain barrier, and their role in intercellular communication make them potential candidates for therapeutic interventions. Studies have indicated that they are effective in wound healing, angiogenesis, neuroprotection, immunomodulation, and treating diseases such as Parkinson’s, gestational diabetes, preeclampsia, and liver and kidney abnormalities.

The diversity of RNA species in exosomes allows for a comprehensive approach to therapy. For example, miRNAs miR-136, miR-335-5p, and miR-1246 have been linked to cardiac repair, renal fibrosis reduction, and hepatic ischaemia-reperfusion injury mitigation, respectively. Similarly, differential expression of circRNAs, lncRNAs, and mRNAs in exosomes from disorders such as preeclampsia and gestational diabetes sheds light on disease mechanisms and prospective treatment targets. Looking ahead, several crucial issues require deeper investigation:

1) Standardization of exosome isolation and characterization: creating standardized techniques for exosome isolation, purification, and characterization would improve reproducibility and speed up clinical translation. 2) Targeted Exosome engineering: creating exosomes with specific RNA payloads or surface changes for targeted transport to sick tissues may improve therapeutic efficacy while reducing off-target effects. 3) Large-scale production and quality control: developing methods for large-scale, GMP-compliant exosome production as well as stringent quality control measures is critical for clinical use. 4) In-depth RNA profiling: Exosomal RNA profiling across illnesses and physiological states can reveal a multitude of biomarkers and therapeutic targets. 5) RNA packaging mechanisms: understanding the mechanisms driving RNA sorting and packaging into exosomes would allow for more accurate control of exosome content. 6) Clinical Trials: developing well-designed clinical trials to evaluate the safety, effectiveness, and long-term consequences of HucMSC-Exos in a variety of illnesses. 7) Combinatorial Therapies: investigating the possibility of combining exosome therapy with conventional medications or other regenerative techniques may result in synergistic results. 8) Personalized Medicine: tailoring exosome therapy to particular patient profiles and illness characteristics may open the way for personalized regenerative medicine.

Finally, the varied RNA composition of HucMSC-Exos provides interesting opportunities for therapeutic interventions across a wide range of illnesses. However, in order to make this potential a reality in the clinic, various problems in exosome synthesis, characterization, and targeted distribution must be addressed. The future of exosome-based therapeutics is bright, with the potential to transform regenerative medicine and customized healthcare.

## Data Availability

Not applicable
